# Men Show Reduced Cardiac Baroreceptor Sensitivity during Modestly Painful Electrical Stimulation of the Forearm: Exploratory Results from a Sham-Controlled Crossover Vagus Nerve Stimulation Study

**DOI:** 10.3390/ijerph182111193

**Published:** 2021-10-25

**Authors:** Elisabeth Veiz, Susann-Kristin Kieslich, Julia Staab, Dirk Czesnik, Christoph Herrmann-Lingen, Thomas Meyer

**Affiliations:** 1 Department of Psychosomatic Medicine and Psychotherapy, University Medical Center, University of Göttingen, 37075 Göttingen, Germany; s.kieslich@stud.uni-goettingen.de (S.-K.K.); julia.staab@med.uni-goettingen.de (J.S.); cherrma@gwdg.de (C.H.-L.); 2 Department of Neurology, University Medical Center, University of Göttingen, 37075 Göttingen, Germany; dczesni@gwdg.de; 3 German Centre for Cardiovascular Research (DZHK), Partner Site Göttingen, 37075 Göttingen, Germany

**Keywords:** spontaneous baroreceptor sensitivity, transcutaneous vagus nerve stimulation, sex differences, heart rate variability, nerve stimulation, stress

## Abstract

This paper presents data from a transcutaneous vagus nerve stimulation experiment that point towards a blunted cardiac baroreceptor sensitivity (cBRS) in young males compared to females during electrical stimulation of the forearm and a rhythmic breathing task. Continuous electrocardiography, impedance cardiography and continuous blood-pressure recordings were assessed in a sex-matched cohort of twenty young healthy subjects. Electrical stimulation of the median nerve was conducted by using a threshold-tracking method combined with two rhythmic breathing tasks (0.1 and 0.2 Hz) before, during and after active or sham transcutaneous vagus nerve stimulation. Autonomic and hemodynamic parameters were calculated, and differences were analyzed by using linear mixed models and post hoc F-tests. None of the autonomic and hemodynamic parameters differed between the sham and active conditions. However, compared to females, male participants had an overall lower total cBRS independent of stimulation condition during nerve stimulation (females: 14.96 ± 5.67 ms/mmHg, males: 11.89 ± 3.24 ms/mmHg, *p* = 0.031) and rhythmic breathing at 0.2 Hz (females: 21.49 ± 8.47 ms/mmHg, males: 15.12 ± 5.70 ms/mmHg, *p* = 0.004). Whereas vagus nerve stimulation at the left inner tragus did not affect the efferent vagal control of the heart, we found similar patterns of baroreceptor sensitivity activation over the stimulation period in both sexes, which, however, significantly differed in their magnitude, with females showing an overall higher cBRS.

## 1. Introduction

Gathering knowledge about inherent differences between men and women is important in cardiovascular medicine, and ongoing research about sex-specific differences in cardiovascular control may have a great impact on the treatment management and outcomes for male and female patients [1,2,3,4,5]. Sex differences in cardiovagal and sympathetic baroreflex functions have been studied by using various assessment methods [6]. Several studies have determined sex differences in the baroreceptor sensitivity in young healthy subjects in supine position during the administration of an intravenous bolus injection of phenylephrine alone or in combination with nitroprusside (modified Oxford method) [7,8,9,10]. These findings suggest that women display lower cardiovagal baroreceptor sensitivity (cBRS) as a response towards drug-induced acute hypertension [6,7,8,9,10]. Similar results were obtained during anesthesia, using phenylephrine [11]. In contrast, another study that used the modified Oxford method in combination with an intravenous slow drug infusion reported no sex differences during slow increase and decrease of blood pressure (BP) [12].

Similarly, spontaneous cBRS during the Valsalva maneuver was comparable between the two sexes during the decrease of BP, but lower for women during the increasing BP phase [13]. Depending on the assessment method used, these results may suggest that BP changes affect cBRS differently between the sexes in young healthy subjects. The sensitivity of the baroreceptors is defined as the ratio of the temporal change between two consecutive R waves (length of RR intervals, where R is defined as the peak of the QRS complex in the electrocardiography (ECG)) in milliseconds per unit change in systolic BP, measured in millimeters of mercury [14]. Spontaneous cBRS can be calculated by using non-invasive techniques, such as the sequence method or the transfer function [14,15,16,17]. Using the sequence method for a large healthy cohort, total cBRS was found to be lower in women than men; however, this was only for age groups under 50 years. Above 50 years, total cBRS was higher within women [18]. Other data also suggest that sex differences in cBRS change upon age, with older subjects showing either no differences or higher cBRS for women [8,17,19].

Since most recordings were performed in supine position and without external stressors, less is known about how spontaneous cBRS differs between young men and women under different stress conditions. This paper presents observational data during a transcutaneous auricular vagus nerve stimulation experiment that hint at a potential lower total cBRS in young men compared to women during and after electrical stimulation of the forearm.

## 2. Materials and Methods

### 2.1. Study Participants

Twenty sex-matched young subjects above 18 years and under 30 years were included in this study. All subjects were required to be healthy and have no underlying somatic or mental diseases. The following experiments were conducted in accordance with the World Medical Association Declaration of Helsinki and were approved by the ethics committee at the University Medical Center Göttingen (number of approved protocol: UMG 27/7/18). All study participants gave their written informed consent on each day of the measurements.

### 2.2. Experimental Protocol

**Figure 1:**
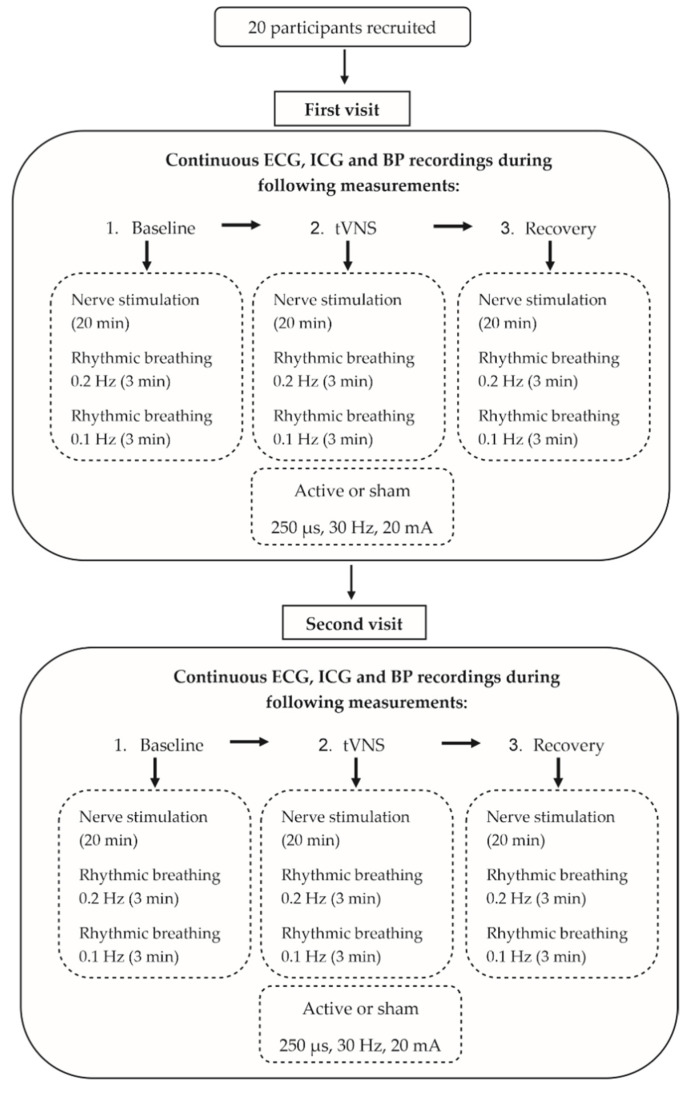
Experimental protocol.

The measurements were performed on two different days, with a minimum wash-out period of 24 h. Continuous ECG, impedance cardiography (ICG) and continuous BP recordings were assessed under changing stimulation conditions (Figure 1). First, median nerve excitability was measured by using the threshold-tracking method for the forearm, followed by a rhythmic breathing exercise before (baseline), during and after (post-stimulation) an active or sham transcutaneous vagus nerve stimulation (tVNS), with each part lasting around thirty minutes. After the experiment, subjects rated their subjective pain during the electrical stimulation on a numeric analog scale (NAS) ranging from 0 to 10. Subjects were told to refrain from caffeine, nicotine and alcohol consumption for at least three hours before each scheduled measurement, and no medication, except for contraceptives, was allowed.

#### 2.2.1. Assessment of cBRS and Other Hemodynamic Parameters

Continuous ECG, ICG and BP recordings were assessed by using the Task Force Monitor (CNSystems, Graz, Austria). Subjects stayed in a relaxed sitting position, with their arms lying on armrests. The skin was cleaned for electrode placement with disinfectant alcohol. Four one-use foam ECG electrodes with a carbon snap (43 × 45 mm, ASF40C, Asmuth, Minden, Germany) were placed on the upper body, and ICG electrodes were placed on the rib cage and neck, as well as a reference electrode above the left shin bone. Oscillatory BP was measured automatically, using a sphygmomanometer cuff on the right arm, while continuous BP was recorded with CNAP finger sensors (CNSystems) on the middle and ring finger of the left hand. The Task Force Monitor software calculated spontaneous cBRS events, using the sequence method. An event describes a sequence in which a monotonic increase or decrease of at least four milliseconds in the RR intervals (RRI), and of at least one mmHg in systolic BP, occurs on three or more consecutive heartbeats. A delay of zero described a synchronization of events in time, whereas a delay of one event occurred when the three consecutive increasing or decreasing BP values of the sequence were delayed by one RRI and a delay of two events occurred if they were delayed by two RRIs, respectively [15,16]. For the analysis, only cBRS events with a delay of one beat were considered. Besides BP, heart rate and cBRS, the normalized high-frequency component of heart rate variability (HF-HRV) was calculated and exported by the software. Additionally, the standard deviation of the RRI (SDNN) and the root mean square of successive differences (RMSSD) were calculated.

#### 2.2.2. Electrical Stimulation of the Median Nerve

Median nerve stimulation was performed by using the threshold-tracking stimulation protocol [20]. With this method, peripheral nerves were electrically stimulated and the compound action potentials of the muscle fibers were tracked. The stimulating electrode (cathode) was placed on the median nerve at the left wrist and the anode ~10 cm proximal at the radial edge of the left forearm. A measurement electrode was placed on the abductor pollicis brevis, and a reference electrode was placed on the proximal phalanx of the left thumb. The grounding electrode was placed on the palm. Before electrode placement, the skin was cleaned by using disinfectant alcohol and roughened slightly by using abrasive paper to increase skin conductance. The electrical stimulation and the muscle reactions are often reported to be uncomfortable and sometimes painful. Therefore, before the measurement, all participants were informed that the application could be painful. Subjects always had the option to demand discontinuation of the measurement.

#### 2.2.3. Rhythmic Breathing Exercises

To control for respiratory sinus arrhythmia, subjects performed a rhythmic breathing exercise after nerve stimulations at a respiratory rate of 0.2 Hz (12 breaths per minute) and subsequently at a rate of 0.1 Hz (6 breaths per minute) for three minutes each. The higher rhythmic breathing rate of 0.2 Hz represents a natural rate in adults and therefore reflects cardiovascular functions under normal breathing conditions. In contrast, the slow-paced breathing at 0.1 Hz maximizes heart rate variability [21] and was included to study potential tVNS effects under additional parasympathetic activation. In order to achieve the rhythmic breathing frequencies, the freely available telephone app “Breathe” (Jatra Ltd., Brighton, UK) was used. Participants were instructed to synchronize their breathing rhythm by visually following a circle moving along a continuous wave line, which was presented on a screen. Additionally, the thickness of the circle changed respectively to represent tidal volume changes. Participants had time to familiarize themselves with the rhythmic breathing task before the measurement started and were instructed to keep their normal breathing depth to prevent hyperventilation.

#### 2.2.4. Transcutaneous Vagus Nerve Stimulation

Each subject received both an active and sham vagus nerve stimulation performed on two different days. On the first day of experiments, the proband was randomized with respect to the stimulation condition (active or sham) executed on that day. Active vagus nerve stimulation was applied at the inner tragus of the left ear, after skin cleaning, skin roughening and application of conductive gel, using the stimulation Easy Tens+ device with customized bipolar clip electrodes (both obtained from body clock, London, UK). The stimulation parameters were set to be biphasic, rectangular pulses with a pulse width of 250 µs, a frequency of 30 Hz and a current intensity of 20 mA. This intensity was felt by the subjects as a tingling sensation, but it was never reported as being painful. During the sham stimulation, the same stimulation protocol was applied, with a change in stimulation site from the left tragus to the left earlobe, due to its lack of vagal nerve innervations [22].

### 2.3. Statistical Data Analysis

ECG data during the conditions of nerve stimulation and during rhythmic breathing were analyzed separately. After artefact and outlier removals, preprocessed data from each subject were standardized with their respective baseline recording before tVNS. Time-series data were linearly detrended over the whole duration, regardless of conditions, to preserve differences by using the Python module emd (version 0.4.0) for empirical mode decomposition [23]. The cardiovascular data were analyzed by using linear mixed models (LMMs) with a custom design matrix to investigate significant changes between the baseline condition and stimulation condition (tVNS), as well as the stimulation condition (tVNS) and the post-stimulation condition within individuals. For the implementation of the LMMs, the Python module statsmodels was used [24]. Sex was additionally included as a fixed main effect to test for sex-specific responses. A group variable was added as a random effect to take variability between subjects into account. A post hoc F-test was used on the LMM coefficients between sham and active to test the joint linear hypotheses of changes in the hemodynamic parameters being only present during the active measurement conditions, but not during the sham recordings. In addition, LMMs were used to test for main sex effects in the baseline characteristics and total cBRS. Unstandardized effect sizes are reported in the form of the estimated LMM coefficients and their 95% confidence intervals. Age and BMI were tested by using the Mann–Whitney U test. Python, Version 3.8.5, was used for all steps of the analysis, including data extraction, data cleaning and statistical analysis. Correction for multiple testing was not conducted after careful deliberation, given the fact that this analysis was part of an exploratory study. Table 1 shows non-standardized values for better visualization and comparison purposes. Figures were created by using the Python modules seaborn (0.11.1) [25] and matplotlib (3.4.2) [26] as well as CoralDraw 2019 (Corel Corporation, Ottowa, Canada).

## 3. Results

### 3.1. Baseline Characteristics

Table 1 shows the baseline characteristics of the total sex-matched study cohort separately for male and female probands during nerve stimulation, as well as during the rhythmic breathing tasks. Male and female study participants did not significantly differ with respect to their age (women: 23.3 ± 1.6, men: 23.4 ± 1.4, *p* = 0.423), BMI (women: 21.1 ± 1.7, men: 22.7 ± 2.3, *p* = 0.081, Table 1) and heart rate during all recorded measurements (nerve stimulation: *p* = 0.803, rhythmic breathing 0.2 Hz: *p* = 0.970, rhythmic breathing 0.1 Hz: *p* = 0.983, Table 1). In all phases of the stimulation protocol, both systolic and diastolic BP were higher for men as compared to women, and significant differences were observed during nerve stimulation and the rhythmic breathing task at 0.2 Hz (nerve stimulation: sBP: *p* = 0.001, dBP: *p* = 0.003, rhythmic breathing 0.2 Hz: sBP: *p* = 0.002, dBP: *p* = 0.002, rhythmic breathing 0.1 Hz: sBP: *p* = 0.059, dBP: *p* = 0.118, Table 1).

### 3.2. Data from Rhythmic Breathing

**Figure 2:**
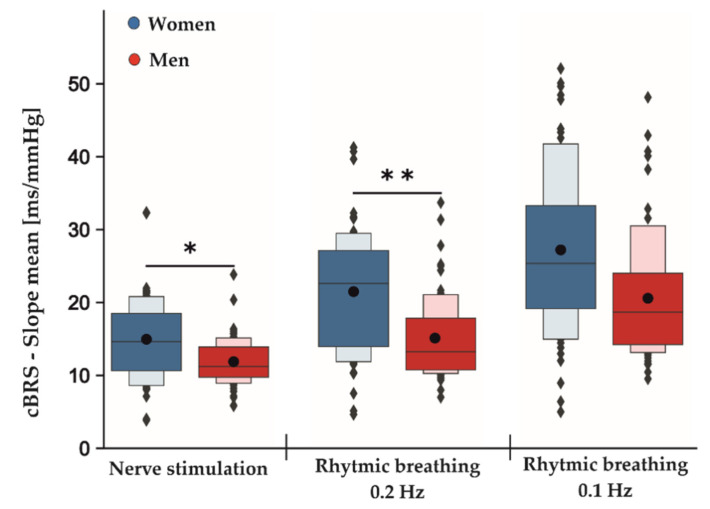
Main sex effects in cBRS during different phases of the experimental protocol independent of active and sham stimulation. Men displayed an overall significant lower total cBRS than women during nerve stimulation and rhythmic breathing at 0.2 Hz and non-significantly at 0.1 Hz. The dark-colored boxes depict 50% of all the data points. Together with the light-colored boxes, they represent 75% of all the measured data. While the black dots represent the means averaged over all conditions (baseline, tVNS and post-stimulation) for the indicated tasks, the gray lines within the boxes show the respective medians; * *p* < 0.05; ** *p* < 0.01.

Table 2 shows the estimated coefficients for all parameters describing the effects between measurement conditions (baseline, tVNS and post-stimulation) and the differences between active and sham stimulations for both rhythmic breathing exercises. The F-test results showed no significant differences between sham and active stimulation conditions in the RMSSD for rhythmic breathing at 0.1 Hz (F_2,113_: 0.059, *p* = 0.943) and at 0.2 Hz (F_2,113_: 0.899, *p* = 0.409). Similarly, neither SDNN (rhythmic breathing 0.1 Hz: F_2,113_: 0.015, *p* = 0.986, rhythmic breathing 0.2 Hz: F_2,113_: 1.789, *p* = 0.172) nor HF-HRV (rhythmic breathing 0.1 Hz: F_2,113_:0.141, *p* = 0.868, rhythmic breathing 0.2 Hz: F_2,113_: 1.472, *p* = 0.234) were significantly different between active and sham stimulation. During the rhythmic breathing task at 0.1 Hz, the sequence method failed to record cBRS events during baseline measurements for two female subjects and for one female subject at 0.2 Hz, respectively. Therefore, these subjects were excluded from the cBRS analysis for tVNS effects, but remained for the analysis of the main sex effect. There were no significant differences in the total cBRS between sham and active stimulation conditions for both rhythmic breathing exercises (rhythmic breathing 0.1 Hz: F_2,107_: 1.596, *p* = 0.208, rhythmic breathing 0.2 Hz: F_2,101_: 1.881, *p* = 0.158). Neither of the rhythmic breathing tasks showed significant sex-specific responses towards the stimulation conditions in any of the tested parameters. However, an overall significant main sex effect was present for the total cBRS during the rhythmic breathing task at 0.2 Hz (*p* = 0.004, Table 3). Here, men showed a significantly lower total cBRS compared to women (women: 21.49 ± 8.47 ms/mmHg, men: 15.12 ± 5.70 ms/mmHg, Figure 2). This effect did not reach significance during the following rhythmic breathing task at 0.1 Hz (women: 27.21 ± 11.12 ms/mmHg, men: 20.58 ± 8.59 ms/mmHg, *p* = 0.061, Figure 2). Although the estimated effect at 0.1 Hz was as high as for the breathing task at 0.2 Hz, its confidence intervals were greater, and this probably led to the insignificant *p*-value (Table 3).

### 3.3. Results from Electrical Stimulation

**Figure 3:**
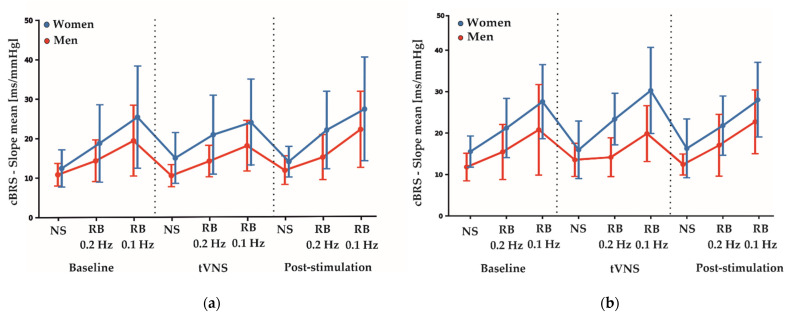
(**a**) Average fluctuations of total cBRS between genders over the time course of the sham measurement day. (**b**) Average fluctuation of total cBRS between genders over the time course of the active measurement day. Depicted are means ± SD for each condition and task. NS: nerve stimulation. RB: rhythmic breathing.

Table 4 shows the estimated coefficients for all parameters describing the effects between measurement conditions (baseline, tVNS and post-stimulation) and the differences between active and sham stimulations during electrical nerve stimulations. Here, the F-test results showed no significant differences between the sham and active stimulation conditions in the RMSSD (F_2,113_: 0.844, *p* = 0.433), SDNN (F_2,113_: 0.476, *p* = 0.623) and HF-HRV (F_2,113_: 0.016, *p* = 0.985) values. Furthermore, total cBRS was similar between sham and active stimulation conditions (F_2,113_: 0.895, *p* = 0.412). There were no significant sex-specific responses to the stimulation conditions found for SDNN, RMSSD, HF-HRV and total cBRS. Notably, similar to the rhythmic breathing task at 0.2 Hz, we found a significant main sex effect for the total cBRS (*p* = 0.031, Table 3), where men showed a significantly lower total cBRS compared to women (women: 14.96 ± 5.67 ms/mmHg, men: 11.89 ± 3.24 ms/mmHg, Figure 2). Figure 3 depicts the fluctuation of total cBRS over the time course of both measurement days, showing that men and women reacted similarly during all conditions with men having lower total cBRS values than women. Interestingly, for both sexes, a linear increase of the average cBRS can be seen consistently between conditions, with the lowest values during the nerve stimulation and the highest values during the slow breathing phases (Figure 2 and Figure 3).

### 3.4. Subjective Pain Ratings during Electrical Stimulation

The NAS scores from one female subject had to be removed before statistical analysis due to a missed NAS rating (Table 5). Subjective pain ratings measured by a numeric analog scale showed no significant differences depending on the stimulation condition (NAS: F_2,107_: 0.686, *p* = 0.506; *Baseline—tVNS*: [active: Coef.: −4.246 (CI: −8.690, 0.198), sham: Coef.: −6.228 (CI: −10.672, −1.784), Diff. in Coeff.: 1.982 (−2.524, 6.488)]; *tVNS—post-stimulation*: [active: Coef.: −0.544 (CI: −4.988, 3.900), sham: Coef.: −4.298 (CI: −8.742, 0.146), Diff. in Coeff.: 3.754 (−0.752, 8.260)]). In addition, there was no significant pain rating difference between men and women (LMM—Intercept (women): Coef.: 40.759 (CI: 28.887, 52.631); sex: Coef.: −4.093 (CI: −20.457, 12.272), *p* = 0.624).

## 4. Discussion

The present study did not detect acute changes in hemodynamic parameters during auricular vagus nerve stimulation at the tragus compared to sham controls. However, we found that spontaneous total cBRS was overall lower in the male study participants as compared to their female counterparts. This effect was significant during both nerve stimulation and the rhythmic breathing task at 0.2 Hz. The same tendency was found during the rhythmic breathing episodes at 0.1 Hz, but this relationship did not reach statistical significance. In addition, average cBRS recurrently showed the tendency to increase between stimulation conditions for both sexes. Nevertheless, we found similar sex-independent patterns of cBRS changes during all conditions.

One current challenge in tVNS research is still the lack of reliable biomarkers that indicate the responsiveness of subjects. Previous studies using stimulation protocols at the tragus or cymba conchae showed mixed results, and there is still no clear conclusion about the usefulness of HRV parameters as biomarkers [27,28,29,30,31,32,33,34]. Various HRV parameters have been evaluated as potential indicators for efferent vagal activation of the heart during transcutaneous cervical as well as auricular tVNS [29]. The results of our study would further suggest that HRV parameters are not suited as reliable markers for acute changes during the application of the current tVNS protocol at the left tragus. Since the effects of auricular tVNS are hypothesized to have mainly afferent effects via the nucleus tractus solitarii [29,35,36], these results do not exclude a potential afferent effectiveness of the current stimulation protocol on other parameters, which have not been tested here. Within this sample, total cBRS did not change significantly under active stimulation compared to sham stimulation. In contrast to this result, two other studies with very similar vagus nerve stimulation protocols found significant increases in total cBRS measured by the sequence method [27,28]. However, Antonino et al. [27] included only men due to possible sex differences, while Bretherton et al. [28] recruited in their study exclusively subjects aged 55 years or older. In addition, although stimulation frequency and pulse width were similar, the used device as well as the stimulation intensity differed from our study. These differences may have contributed to these opposing results.

The main observation in the present study was the overall lower cBRS in men as compared to their female counterparts. Since this reduction was present during all stimulation conditions and the responses between stimulation conditions were similar for both sexes, this observation was not a result of the vagus nerve stimulation. The overall higher cBRS observed in female probands than that in males might be related to their distinct reaction towards a potential stressful situation. Firstly, the obligatory preannouncement that the electrical stimulation of the forearm during the recording might be uncomfortable and even painful imposed the risk of psychological stress. Secondly, all experiments were conducted by a young female experimenter, which might have had a greater influence on the psychological reactions of male participants, since all subjects had to partially undress for the applications of the ECG electrodes, and previous studies had shown that the gender of the examiner can affect certain outcomes of experiments, such as the subjective pain ratings [37,38,39,40,41]. Interestingly, the subjective pain ratings obtained after the current application did not show any significant differences depending on gender. Furthermore, it was found that, in mentally stressful experiments, men reacted with a significantly higher systolic BP than women [42,43,44,45]. In our study, men also had higher systolic BP values than women, and this may be linked to their overall lower cBRS. Similar cBRS changes in both sexes with time, the highest increase occurring during slow breathing at 0.1 Hz, suggest that we indeed measured an important component of the vagal tone during all stimulation conditions. Due to our study protocol, it is not possible to unveil the actual cause of these findings.

This observation seems to contradict the results of previous studies using drug-induced BP changes [6,7,8,9,10], but it is in line with the results from the study by Klassen et al. [46], who, using the transfer function method, reported similar results with spontaneous cBRS being higher in females. Furthermore, two recent studies have reported enhanced cBRS in men compared to women during post-exercise ischemia and passive limb movements [47,48]. The nerve stimulation of the median nerve caused passive twitching of the participants’ thumbs. In accordance with the discussion in Klassen et al. [46], a comparison between results of pharmacologically induced BP changes and spontaneously measured cBRS may not be appropriate due to the underlying differences in methods and some evidence that spontaneous cBRS does not reflect experimentally induced cBRS [46,49]. Because in our study spontaneous cBRS was recorded exclusively under mild stress conditions, no conclusion can be drawn about changes in BP and heart rate in situations of extreme stress exposure.

Our findings should be interpreted in the light of several limitations that are inherently present in our study and prohibit any causal interpretation. Important limitations in our study are the lack of neuroimaging techniques and measurements of norepinephrine release. With regard to the statistical methodology, the distributional assumptions for most LMM residuals were not fully met, since they were often slightly skewed. However, simulation studies showed that the estimates under these violations are still quite robust and unbiased, though they may be less precise [50,51]. Furthermore, this study solely assessed cardiac vagal activity and therefore lacks a correlation and comparison with other potential markers for tVNS. Other proposed methods to quantify vagal activity include neuroimaging techniques and measurements of the norepinephrine release [29]. While one study reported an increase in pupil dilation during tVNS, which was not observed in sham stimulation [52], another study demonstrated an increased pupil size only for the left eye under specific scotopic illumination and stimulation conditions [53]. In contrast, two recent studies did not find any changes in pupillary responses and cardiac vagal activity associated with active tVNS during cognitive tasks [54,55]. Using electroencephalography, one study showed an increased attenuation of alpha oscillations during tVNS [53], while another reported a greater power in the delta band frequencies [56].

In addition, there are some limitations regarding the sequence method used for measuring spontaneous cBRS. In our study, the emphasis of the analysis was laid on the vagal cardiac components of the baroreflex due to vagus nerve stimulation. Therefore, any observations should be strictly separated from sympathetic BRS, which usually is quantified by muscle sympathetic nerve activity. A recently published study showed that the sequence method only quantifies the high-frequency respiratory components of the baroreflex, whereas the low-frequency sympathetically modulated components are relatively neglected [57]. Interestingly, the correlation between these two cBRS components also exhibits potential sex differences [58]. In addition, other findings claim that the sequence method may be methodologically biased and cannot quantify the causal relationship between BP and heart rate, but rather expresses the heart-rate-to-blood-pressure variability ratio [59]. The authors argue that this bias may mainly be a problem in the presence of shallow breathing, which causes only small respiratory-induced BP oscillations that cannot be reliably quantified by the sequence method. In our study, shallow breathing can be virtually excluded for the slow breathing task, since tidal volumes need to increase in order to compensate for the reduced frequency. Strengths of this study are the crossover design, the continuous recording of physiological variables and the use of standardized paced-breathing, which reduced inter-individual differences and controlled respiratory effects on the autonomic and cardiovascular control.

## 5. Conclusions

In summary, our study demonstrated that the auricular vagus nerve stimulation at the left inner tragus did not increase the efferent vagal control of the heart, leading to further evidence that HRV parameters may not be suited as reliable tVNS markers in young healthy subjects. The lack of assessment of other potential markers is a relevant limitation in this study, which requires future investigations, using multiple neurophysiological and autonomic measurements to compare various tVNS markers, as well as different stimulation protocols. Nevertheless, we found that the total cBRS was lower in men compared to women, after the preannouncement of a potentially uncomfortable and painful electrical stimulation, while subjective pain ratings were unaltered. This observation supports evidence for cardiovagal sex differences upon stress application. More studies with larger sample sizes are needed to verify these findings. In addition, it would be of great interest to investigate the effects of different types of stressors and whether stress-related gender differences in cBRS can also be found in the older population. Since cBRS in women may be affected by their menstrual cycle and menopause, the relationship with these influencing factors should be systematically examined. Although this study was based only on a small sample size, our findings are useful for planning future experiments on the sex-specific effects of psychological stressors on cardiovascular functions.

## Figures and Tables

**Table 1:** Baseline characteristics of participants during nerve stimulation and rhythmic breathing before vagus nerve stimulation. Displayed are means ± SD. Baseline value differences between sexes were tested by using Mann–Whitney U test (age and BMI) and linear mixed models (heart rate, systolic blood pressure (sBP) and diastolic blood pressure (dBP)). Estimated coefficients (Coeff.), representing the effect of being male, and their respective 95% confidence interval (CI) are depicted in the last column.

Nerve Stimulation	Stimulation	Total Cohort (*n* = 20)	Women (*n* = 10)	Men (*n* = 10)	Coeff. (CI-95) Sex Effect—Men
Age (years)		23.35 ± 1.46	23.30 ± 1.64	23.40 ± 1.35	
BMI (kg/cm^2^)		21.90 ± 2.12	21.13 ± 1.65	22.67 ± 2.33	
Heart rate (bpm)	active	75.57 ± 7.95	74.87 ± 9.46	76.27 ± 6.54	1.098 (−7.543, 9.738)
	sham	76.43 ± 12.58	76.04 ± 16.55	76.83 ± 7.74	
sBP (mmHg)	active	101.87 ± 10.61	95.96 ± 9.38	107.79 ± 8.47	10.859 (4.372, 17.346)
	sham	104.67 ± 11.04	99.73 ± 11.00	109.61 ± 9.04	
dBP (mmHg)	active	62.56 ± 9.40	58.07 ± 8.44	67.05 ± 8.41	9.300 (3.155, 15.445)
	sham	63.37 ± 10.18	58.56 ± 10.02	68.18 ± 8.20	
**Rhythmic breathing 0.1 Hz**					
Heart rate (bpm)	active	74.73 ± 7.26	74.43 ± 7.92	75.03 ± 6.96	−0.085 (−7.996, 7.825)
	sham	74.66 ± 11.99	75.04 ± 15.93	74.27 ± 7.03	
sBP (mmHg)	active	101.21 ± 8.55	97.76 ± 8.02	104.67 ± 7.97	7.343 (−0.276, 14.962)
	sham	107.94 ± 12.23	104.05 ± 11.53	111.83 ± 12.22	
dBP (mmHg)	active	64.33 ± 6.93	61.27 ± 7.96	67.40 ± 4.15	4.240 (−1.071, 9.551)
	sham	67.56 ± 8.72	66.38 ± 10.08	68.73 ± 7.47	
**Rhythmic breathing 0.2 Hz**					
Heart rate (bpm)	active	76.84 ± 8.17	76.34 ± 9.69	77.33 ± 6.81	0.169 (−8.662, 9.000)
	sham	76.02 ± 12.82	76.34 ± 16.60	75.69 ± 8.44	
sBP (mmHg)	active	106.30 ± 9.90	101.40 ± 10.69	111.21 ± 6.28	10.878 (4.128, 17.629)
	sham	109.86 ± 12.22	103.88 ± 9.48	115.83 ± 12.09	
dBP (mmHg)	active	66.34 ± 8.21	61.65 ± 8.79	71.03 ± 4.01	8.659 (3.280, 14.038)
	sham	69.43 ± 9.09	65.45 ± 8.53	73.40 ± 8.16	

**Table 2:** Estimated linear mixed model coefficients (Coeff.) with their respective 95% confidence interval (CI-95) representing the effect of change between baseline and tVNS, as well as tVNS and post-stimulation during the rhythmic breathing tasks at 0.2 and 0.1 Hz. In addition, the differences (Diff.) in coefficients (active minus sham) are displayed with their respective CI-95. s.u. = baseline standardized unit.

Coefficient (CI-95)	Baseline—tVNS		tVNS—Post-Stimulation	
	Active	Sham	Active	Sham
**Rhythmic breathing 0.2 Hz**				
HF-HRV (s.u.)	0.012 (−0.085, 0.110)	−0.028 (−0.125, 0.07)	−0.073 (−0.171, 0.025)	0.006 (−0.092, 0.104)
Diff. in Coeff.	0.04 (0.038, 0.042)		−0.079 (−0.081, −0.077)	
RMSSD (ms)	−0.561 (−3.694, 2.57)	0.772 (−2.360, 3.905)	1.520 (−1.612, 4.653)	−0.171 (−3.303, 2.961)
Diff. in Coeff.	−1.333 (−3.571, 0.906)		1.691 (−0.548, 3.930)	
SDNN (ms)	2.346 (−1.105, 5.798)	2.680 (−0.772, 6.132)	3.550 (0.098, 7.002)	−0.353 (−3.805, 3.099)
Diff. in Coeff.	−0.334 (−3.053, 2.385)		3.903 (1.184, 6.622)	
cBRS (s.u.)	0.055 (−0.226, 0.336)	0.361 (0.080, 0.642)	0.057 (−0.224, 0.338)	0.424 (0.143, 0.704)
Diff. in Coeff.	−0.306 (−0.324, −0.288)		−0.367 (−0.385, −0.349)	
**Rhythmic breathing 0.1 Hz**				
HF-HRV (s.u.)	0.047 (−0.017, 0.111)	0.025 (−0.039, 0.089)	0.010 (−0.054, 0.074)	0.008 (−0.056, 0.072)
Diff. in Coeff.	0.022 (0.021, 0.023)		0.002 (0.001, 0.003)	
RMSSD (ms)	1.666 (−2.593, 5.925)	2.548 (−1.712, 6.807)	2.220 (-2.039, 6.480)	2.157 (−2.102, 6.416)
Diff. in Coeff.	−0.882 (−5.022, 3.258)		0.063 (−4.077, 4.203)	
SDNN (ms)	2.989 (−2.301, 8.278)	2.337 (−2.952, 7.627)	3.459 (-1.830, 8.748)	3.105 (−2.184, 8.394)
Diff. in Coeff.	0.652 (−5.732, 7.036)		0.354 (−6.030, 6.738)	
cBRS (s.u.)	0.187 (−0.074, 0.448)	0.213 (−0.048, 0.474)	0.129 (-0.133, 0.390)	0.432 (0.171, 0.693)
Diff. in Coeff.	−0.026 (−0.042, −0.011)		−0.303 (−0.319, −0.288)	

**Table 3:** Main sex effects of total cardiac baroreceptor sensitivity (cBRS). Results of linear mixed models are shown. Women are included in the Intercept, while males are represented in the variable “Sex”.

Total cBRS		Coefficient (CI-95%)	*p*-Value
Nerve stimulation	Intercept	14.956 (12.982, 16.929)	
	Sex	−3.070 (−5.862, −0.279)	0.031
Rhythmic breathing 0.1 Hz	Intercept	27.215 (22.296, 32.133)	
	Sex	−6.637 (−13.593, 0.318)	0.061
Rhythmic breathing 0.2 Hz	Intercept	21.609 (18.442, 24.775)	
	Sex	−6.492 (-10.961, −2.023)	0.004

**Table 4:** Estimated linear mixed model coefficients (Coeff.) with their respective 95% confidence interval (CI-95) representing the effect of change between baseline and tVNS, as well as tVNS and post-stimulation during electrical nerve stimulation. Additionally, each difference (Diff.) in coefficients (active minus sham) is shown including the respective CI-95; s.u. = baseline standardized unit.

Coefficient (CI)	Baseline—tVNS		tVNS—Post-Stimulation	
	Active	Sham	Active	Sham
**Electrical nerve stimulation**				
HF-HRV (s.u.)	0.017 (−0.038, 0.072)	0.023 (−0.032, 0.077)	0.026 (−0.029, 0.081)	0.032 (−0.023,0.087)
Diff. in Coeff.	−0.006 (−0.007, −0.005)		−0.006 (−0.007, 0.005)	
RMSSD (ms)	0.961 (−1.721, 3.643)	3.032 (0.350, 5.714)	0.262 (−2.420, 2.944)	2.533 (−0.149, 5.215)
Diff. in Coeff.	−2.071 (−3.712, −0.430)		−2.271 (−3.912, −0.630)	
SDNN (ms)	2.293 (−0.803, 5.389)	3.227 (0.131, 6.323)	0.923 (−2.173, 4.019)	3.094 (−0.002, 6.190)
Diff. in Coeff.	−0.934 (−3.121, 1.253)		−2.171 (−4.358, 0.016)	
cBRS (s.u.)	0.087 (−0.089, 0.263)	0.194 (0.018, 0.370)	−0.018 (−0.194,0.157)	0.149 (−0.027,0.325)
Diff. in Coeff.	−0.107 (−0.114, −0.100)		−0.167 (−0.174, −0.160)	

**Table 5:** Subjective pain rating scores, using the numeric analog scale during nerve stimulation. Depicted are means ± SD on the NAS, as measured in millimeters (ranging from 0 to 100).

	Numeric Analog Scale (NAS)	Men (*n* = 10)	Women (*n* = 9)	Total (*n* = 19)
Active	Baseline	39.70 ± 23.09	49.89 ± 14.28	44.53 ± 19.61
	tVNS	32.70 ± 23.91	40.89 ± 19.37	36.58 ± 21.69
	Post-stimulation	36.30 ± 21.26	43.56 ± 20.88	39.74 ± 20.83
Sham	Baseline	45.80 ± 26.08	40.22 ± 15.82	43.16 ± 21.44
	tVNS	32.60 ± 22.21	37.67 ± 20.81	35.00 ± 21.12
	Post-stimulation	32.90 ± 19.44	32.33 ± 23.12	32.63 ± 20.66

## Data Availability

The datasets generated during and/or analyzed during the current study are available from the corresponding author on reasonable request.
